# Racial and Ethnic Disparities in Patient Restraint in Emergency Departments by Police Transport Status

**DOI:** 10.1001/jamanetworkopen.2024.0098

**Published:** 2024-02-21

**Authors:** Erika Chang-Sing, Colin M. Smith, Jane P. Gagliardi, Laura D. Cramer, Leah Robinson, Dhruvil Shah, Morgan Brinker, Patelle Jivalagian, Yue Hu, Nicholas A. Turner, Ambrose H. Wong

**Affiliations:** 1 Yale School of Medicine, New Haven, Connecticut; 2 Hubert-Yeargan Center for Global Health, Duke University, Durham, North Carolina; 3 Department of Psychiatry and Behavioral Sciences, Duke University School of Medicine, Durham, North Carolina; 4 Department of Medicine, Duke University School of Medicine, Durham, North Carolina; 5 Yale National Clinician Scholars Program, New Haven, Connecticut; 6 Department of Emergency Medicine, Yale School of Medicine, New Haven, Connecticut; 7 Yale School of Public Health, New Haven, Connecticut

## Abstract

**Importance:**

Black patients are more likely than White patients to be restrained during behavioral crises in emergency departments (EDs). Although the perils of policing mental health for Black individuals are recognized, it is unclear whether or to what extent police transport mediates the association between Black race and use of physical restraint in EDs.

**Objective:**

To evaluate the degree to which police transport mediates the association between Black race and use of physical restraint in EDs.

**Design, Setting, and Participants:**

This retrospective, cross-sectional study used electronic health record data from ED visits by adults (aged ≥18 years) to 3 hospitals in the southeastern US and 10 in the northeastern US between January 1, 2015, and December 31, 2022. Data were analyzed from September 1, 2022, to May 30, 2023.

**Exposures:**

Race, ethnicity, and police transport to the hospital.

**Main Outcomes and Measures:**

The primary outcome variable was the presence of an order for restraints during an ED visit.

**Results:**

A total of 4 263 437 ED visits by 1 257 339 patients (55.5% of visits by female and 44.5% by male patients; 26.1% by patients 65 years or older) were included in the study. Black patients accounted for 27.5% of visits; Hispanic patients, 17.6%; White patients, 50.3%; and other or unknown race or ethnicity, 4.6%. In models adjusted for age, sex, site, previous behavioral or psychiatric history, and visit diagnoses, Black patients were at increased odds of experiencing restraint compared with White patients (adjusted odds ratio [AOR], 1.33 [95% CI, 1.28-1.37]). Within the mediation analysis, Black patients had higher odds of being brought to the hospital by police compared with all other patients (AOR, 1.38 [95% CI, 1.34-1.42]). Patients brought to the ED under police transport had increased odds of experiencing restraint compared with all other modes of transport (AOR, 5.51 [95% CI, 5.21-5.82]). The estimated proportion of use of restraints for Black patients mediated by police transport was 10.70% (95% CI, 9.26%-12.53%).

**Conclusions and Relevance:**

In this cross-sectional study of ED visits across 13 hospitals, police transport may have mediated the association between Black race and use of physical restraint. These findings suggest a need to further explore the mechanisms by which transport to emergency care may influence disparate restrictive interventions for patients experiencing behavioral emergencies.

## Key Points

**Question:**

To what degree might police transport account for racial and ethnic disparities in use of physical restraint in emergency departments (EDs)?

**Findings:**

This cross-sectional study found that an estimated 11% of the racial and ethnic disparity in physical restraint use experienced by non-Hispanic Black patients in EDs may have been mediated by police transport.

**Meaning:**

The ways in which patients are transported to EDs may be associated with racially and ethnically disparate use of physical restraint.

## Teaser

This cross-sectional study assesses the extent to which police transport mediates the association between Black race and use of physical restraint among patients in emergency departments (EDs) across 2 geographically distinct regions in the US.

## Introduction

Physical restraint use in the emergency setting is prevalent in the US, particularly among patients experiencing behavioral health crises,^1^ despite clinical guidelines recommending restraint use only after less restrictive interventions have failed or in the presence of imminent danger.^2,3^ Physical restraints are applied to prevent patients in behavioral crises or with symptoms of psychomotor agitation from hurting themselves and others.^2,3^ However, restraints are associated with lasting distress, damage the patient-clinician relationship, and sow distrust in the medical system.^3,4^ Various studies have shown that Black patients are more likely to be restrained in emergency settings than White patients, even after controlling for other demographic and clinical factors.^5,6^

Despite growing recognition that racial and ethnic inequities exist in restraint use, we still need to understand the underlying mechanisms driving these outcomes. Among the many factors potentially contributing to restraint use in emergency settings, prehospital interaction with law enforcement may be especially relevant. Recent studies have demonstrated the prevalent use of police in medical transport, particularly among patients with mental illness, and increased morbidity and mortality associated with police interactions for Black patients in the US. ^7,8^

Although mediation analyses in cross-sectional data cannot demonstrate causality, they may assist in estimating the extent to which an exposure may influence an outcome and thereby help elucidate mechanisms that drive health inequities and inform relevant policies and interventions.^9^ The objective of this study was to assess the extent to which police transport may mediate the association between Black race and physical restraint use in emergency departments (EDs) across 2 geographically distinct regions in the US.

## Methods

### Study Design

This retrospective, cross-sectional study evaluated the characteristics of physical restraint use using data from electronic health records. Data were collected for each ED visit between January 1, 2015, and December 31, 2022, among patients 18 years and older presenting to 1 of 3 hospital EDs in a regional hospital network in the southeastern US and 10 hospital EDs in a regional hospital network in the northeastern US. The 3 southeastern US hospitals included an academic level I trauma center, an academic regional hospital, and a community hospital. Hospitals in the northeastern US included 2 nonacademic urban, 2 academic urban, 1 pediatric urban, and 5 nonacademic suburban sites. This study followed the Strengthening the Reporting of Observational Studies in Epidemiology (STROBE) reporting guideline and A Guideline for Reporting Mediation Analyses of Randomized Trials and Observational Studies (AGReMA). The Yale University and Duke University Health System Institutional Review Boards approved this study and waived the need for informed consent owing to the use of deidentified medical record data.

Demographic and visit information—including race and ethnicity, age, sex, history of behavioral diagnoses, and visit diagnoses—were extracted directly from each patient’s electronic health record as entered during the patient encounter. Classifications may represent self-identification or assignment by hospital registration. The primary exposure of interest was race and ethnicity, which was categorized as Black, Hispanic or Latino, White, other race or ethnicity (including American Indian or Alaska Native, Asian, Native Hawaiian or Other Pacific Islander, multiracial, and other race or ethnicity), and unknown race or ethnicity, as documented in the electronic health record. We categorized patients into 6 age groups: 18 to 25, 26 to 35, 36 to 45, 46 to 55, 56 to 64, and 65 years or older, consistent with prior studies.^10^ We grouped relevant past psychiatric and behavioral diagnoses according to the Agency for Healthcare Research and Quality clinical classification software.^11^ We also collected ED diagnosis codes based on *International Statistical Classification of Diseases and Related Health Problems, Tenth Revision*, then categorized them as medical, psychiatric, substance use related, neurological, or traumatic, based on previously described methods.^12^ These diagnosis groups were not mutually exclusive, and each category included cases that involved police transport and physical restraint (ie, patients with medical, neurological, traumatic, substance-related, and psychiatric complaints who were brought to the hospital by police and restrained in the ED).

### Main Outcomes and Measures

The primary outcome variable of interest was the placement of an order for violent physical restraints, indicated for management of behavior that jeopardized the immediate physical safety of the patient, staff, or others as defined by Joint Commission standards.^13^ Orders for nonviolent restraints for protection of equipment or promotion of medical healing, such as preventing self-extubation, were not included in the primary outcome. Violent restraints were broadly defined by their intent of use for violent behavior in behavioral emergencies and may encompass different types of equipment or restraints that overlap with equipment used in nonviolent restraints. The mediator variable of interest was presence of police transport to the ED, standardly reported at triage across included hospital systems. A patient was considered to be transported by police when they arrived at the hospital accompanied by police.

### Statistical Analysis

Descriptive statistics were used to summarize patient demographics, frequency of police transport, and frequency of restraint use. These numbers were calculated using the flextable package in R software, version 4.2.0-foss-2020b (R Project for Statistical Computing).^14^

Each of our regression models was clustered by patient and adjusted for suspected confounders of race and ethnicity, sex, age, site, previous behavioral diagnosis, substance use diagnosis, and diagnosis category for the visit. To assess the robustness of our findings, we then performed a sensitivity analysis by including police transport in our full regression models. All tests were 2 tailed, and *P* < .05 was considered significant. Analysis was completed using the geepack package in R software, version 4.2.0-foss-2020b (R Project for Statistical Computing).^15^ Data analysis for the study occurred from September 1, 2022, to May 30, 2023.

**Figure 1.**
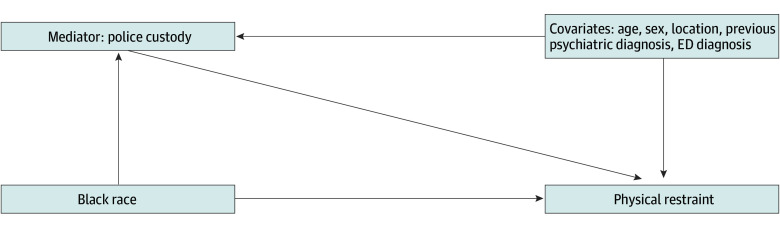
Preliminary Conceptual Model for Mediation of Physical Restraint of Black Patients by Police Transport ED indicates emergency department.

A preliminary conceptual model for our mediation analysis is presented in Figure 1. We included age, sex, location, previous psychiatric diagnoses, and ED diagnosis group (including substance use) as covariates in the directed graph. Demographically, both male sex and ages 18 to 35 years were positively associated with both police transport (mediator) and restraint (outcome) within our data. Diagnostically, patients with previous psychiatric diagnoses, patients with psychiatric ED diagnoses, and patients with substance-related ED diagnoses were at higher odds of being brought to the hospital by police and restrained.

For our mediation analysis, we applied the framework developed by Baron and Kenny^16^ and first developed logistic regression models to measure the association between (1) the exposure (Black race) and the outcome (physical restraint), (2) the exposure (Black race) and the hypothesized mediator (police transport status), and (3) the mediator (police transport status) and the outcome (physical restraint). We then reported the percentage of physical restraint mediated by police transport status using the Mediation package in R software, version 4.2.0-foss-2020b (R Project for Statistical Computing).^17^ We calculated the variance and constructed 95% CI estimates using the bootstrap method.

In this study, we attempted to reduce bias by including EDs in multiple hospital types, by studying 2 distinct geopolitical regions, and by including all adult ED visits with complete data. We attempted to address bias by clustering our models by patient, adjusting our multivariable regressions for possible confounding variables. To assess possible interaction effects, we conducted a post hoc exploration of interactions among race and ethnicity, sex, and age.

## Results

**Figure 2.**
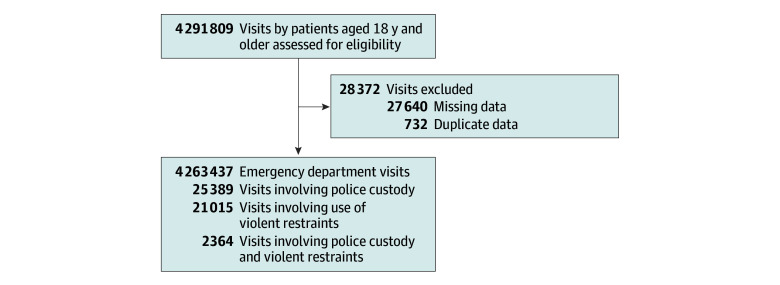
Flow Diagram for Eligible Emergency Department Visits and Those With Outcomes of Interest Included in Study

A total of 4 263 437 ED visits by 1 257 339 patients were included in the study (55.5% of visits by female and 44.5% by male patients; 26.1% of visits by patients 65 years or older). Black patients accounted for 27.5% of visits; Hispanic patients, 17.6%; White patients, 50.3%; and patients of other or unknown race or ethnicity, 4.6% (Table 1). Of ED visits included, 875 985 visits took place at 1 of the 3 hospitals in the southeastern US, and 3 387 452 visits took place at 1 of the 10 hospitals in the northeastern US. Approximately 0.5% of all visits included an order for violent physical restraint (21 015 of 4 263 437). White patients accounted for 2 144 097 visits and 9856 cases of use of restraint (46.9%). Black patients accounted for 1 170 417 visits and 6916 cases of use of restraint (32.9%). Hispanic or Latino patients accounted for 751 942 visits and 3410 cases of use of restraint (16.2%). Of the 25 389 visits in which patients were transported to the hospital by police, 2364 resulted in use of restraint. A total of 732 visits were excluded after they were found to be duplicates by matching visit identification numbers and other variables, and 27 640 visits were excluded from this analysis due to missing data regarding exposures, outcomes, and confounding variables of interest. In the 27 640 visits excluded due to missing diagnosis data, there were 223 cases of restraint (0.8%) and 511 cases of police custody arrival (1.8%) (Figure 2).

**Table 1.** Demographic and Clinical Characteristics of Emergency Department Visits by Presence of Violent Physical Restraint, 2015 to 2022

Characteristic	Patient visits, No. (%) (N = 4 263 437)	
	No violent physical restraint (n = 4 242 422)	Use of violent physical restraint (n = 21 015)
Sex		
Female	2 356 892 (55.6)	7256 (34.5)
Male	1 885 530 (44.4)	13 759 (65.5)
Race and ethnicity		
Black	1 163 501 (27.4)	6916 (32.9)
Hispanic or Latino	748 532 (17.6)	3410 (16.2)
White	2 134 241 (50.3)	9856 (46.9)
Other^a^	148 027 (3.5)	583 (2.8)
Unknown	48 121 (1.1)	250 (1.2)
Age, y		
18-25	563 156 (13.3)	3364 (16.0)
26-35	708 616 (16.7)	5477 (26.1)
36-45	615 611 (14.5)	3574 (17.0)
46-55	673 064 (15.9)	4193 (20.0)
56-64	573 038 (13.5)	2095 (10.0)
≥65	1 108 937 (26.1)	2312 (11.0)
Site		
Northeastern US	3 369 065 (79.4)	18 387 (87.5)
Southeastern US	873 357 (20.6)	2628 (12.5)
Previous psychiatric diagnoses		
No	2 331 459 (55.0)	3287 (15.6)
Yes	1 910 963 (45.0)	17 728 (84.4)
Medical visit diagnoses^b^		
No	1 035 153 (24.4)	12 781 (60.8)
Yes	3 207 269 (75.6)	8234 (39.2)
Psychiatric visit diagnoses^b^		
No	3 973 994 (93.7)	11 350 (54.0)
Yes	268 428 (6.3)	9665 (46.0)
Substance visit diagnoses^b^		
No	3 957 643 (93.3)	7582 (36.1)
Yes	284 779 (6.7)	13 433 (63.9)
Trauma visit diagnoses^b^		
No	3 642 596 (85.9)	19 017 (90.5)
Yes	599 826 (14.1)	1998 (9.5)
Cognitive or neurological visit diagnoses^b^		
No	3 694 719 (87.1)	16 102 (76.6)
Yes	547 703 (12.9)	4913 (23.4)
Police transport		
No	4 219 397 (99.5)	18 651 (88.8)
Yes	23 025 (0.5)	2364 (11.2)

^a^ Includes American Indian or Alaska Native, Asian, Native Hawaiian or Other Pacific Islander, multiracial, and other race or ethnicity.

^b^ Emergency department visit diagnoses were based on *International Statistical Classification of Diseases and Related Health Problems, Tenth Revision* codes, then categorized based on previously described methods.^12^

Within a fully adjusted model for restraint use (Table 2), Black patients were at increased odds of being restrained compared with White patients (adjusted odds ratio [AOR], 1.33 [95% CI, 1.28-1.37]). Hispanic or Latino patients were significantly less likely to be restrained compared with White patients (AOR, 0.93 [95% CI, 0.80-0.96]; *P* < .001). Other factors associated with higher odds of restraint in this study included male sex, age 18 to 35 years, a previous behavioral diagnosis, and psychiatric, substance-related, or neurological diagnoses for the ED visit. Sensitivity analyses with inclusion of police transport in our fully adjusted model for physical restraint use indicated model stability (eTable 1 in Supplement 1). In a post hoc analysis assessing interaction of race and ethnicity, sex, and age, we found that male sex consistently interacted with racial and ethnic minority groups (ie, the racial disparities in restraint rates were greater for male patients than for female patients) (eTable 2 in Supplement 1). Sex and racial disparities were most prominent in the youngest groups and narrowed with older age (eFigure in Supplement 1).

**Table 2.** Multivariable Logistic Regression Analyses for Associations Between Demographic and Visit Characteristics and Violent Restraint Use

Characteristic	Nested unadjusted analysis		Nested adjusted analysis^a^	
	OR (95% CI)	*P* value	OR (95% CI)	*P* value
Sex				
Female	1 [Reference]	NA	1 [Reference]	NA
Male	2.37 (2.30-2.44)	<.001	1.53 (1.48-1.58)	<.001
Race and ethnicity				
Black	1.29 (1.25-1.33)	<.001	1.33 (1.28-1.37)	<.001
Hispanic or Latino	0.99 (0.95-1.03)	.49	0.93 (0.89-0.96)	<.001
White	1 [Reference]	NA	1 [Reference]	NA
Other^b^	0.85 (0.78-0.93)	<.001	1.16 (1.06-1.27)	<.001
Unknown	1.12 (0.99-1.28)	.07	1.73 (1.52-1.97)	<.001
Age, y				
18-25	0.88 (0.84-0.92)	<.001	1.25 (1.19-1.31)	<.001
26-35	1.13 (1.09-1.18)	<.001	1.24 (1.18-1.29)	<.001
36-45	1 [Reference]	NA	1 [Reference]	NA
46-55	0.78 (0.75-0.82)	<.001	0.71 (0.68-0.75)	<.001
56-64	0.54 (0.51-0.57)	<.001	0.57 (0.47-0.61)	<.001
≥65	0.31 (0.29-0.32)	<.001	0.62 (0.59-0.65)	<.001
Previous psychiatric diagnoses				
No	1 [Reference]	NA	1 [Reference]	NA
Yes	6.58 (6.34-6.83)	<.001	2.42 (2.31-2.52)	<.001
Medical visit diagnoses	0.21 (0.20-0.21)	<.001	0.45 (0.43-0.46)	<.001
Psychiatric visit diagnoses	12.61 (12.27-12.96)	<.001	3.62 (3.50-3.75)	<.001
Substance visit diagnoses	24.62 (23.93-25.33)	<.001	9.29 (8.94-9.65)	<.001
Trauma visit diagnoses	0.64 (0.61-0.67)	<.001	1.03 (0.99-1.09)	.17
Cognitive or neurological visit diagnoses	2.06 (1.99-2.13)	<.001	2.09 (2.02-2.16)	<.001

Abbreviations: NA, not applicable; OR, odds ratio.

^a^ Adjusted for sex, race and ethnicity, age, site, previous psychiatric diagnoses, and emergency department visit diagnoses.

^b^ Includes American Indian or Alaska Native, Asian, Native Hawaiian or Other Pacific Islander, multiracial, and other race or ethnicity.

In our mediation analysis (Table 3), the AOR of Black race with restraint, compared with the rest of the population, was found to be 1.33 (95% CI, 1.29-1.37). Next, the AOR of Black race with police transport was 1.38 (95% CI, 1.34-1.42) compared with the rest of the population. The AOR of police transport with restraint was 5.51 (95% CI, 5.21-5.82) compared with those who were not brought in by the police. As these numbers together indicate that the proposed mediator (police transport) had a positive association with both Black race and higher rates of restraint, we proceeded with the mediation analysis. An estimated 10.70% (95% CI, 9.26%-12.53%) of the racial and ethnic disparity in physical restraint use experienced by non-Hispanic Black patients was mediated by police transportation. All values were found to be significant at *P* < .001. This mediation analysis was completed while adjusting for the full set of potential confounding variables (age, sex, place, previous behavioral diagnosis, and ED diagnosis type, including substance use).

**Table 3.** Mediation Analysis of Police Transport Status for Black Race and Use of Physical Restraint for Violence^a^

Assessment for mediation	Unadjusted analysis	Mediation analysis
Total effect estimate for effect of Black race on restraint compared with Hispanic ethnicity, White race, and other race or ethnicity^b^	1.30 (1.26-1.34)	1.33 (1.29-1.37)
Association between Black race and police transport (mediator) compared with Hispanic ethnicity, White race, and other race or ethnicity^b^	1.79 (1.75-1.84)	1.38 (1.34-1.42)
Association between police transport (mediator) and restraint compared with nonpolice transport	23.23 (22.21-24.29)	5.51 (5.21-5.82)
Quantifying mediation effect, % mediated	25.23 (22.30-28.52)	10.70 (9.26-12.53)

^a^ Data are expressed as odds ratios (95% CIs). *P* < .001 for all comparisons.

^b^ Includes American Indian or Alaska Native, Asian, Native Hawaiian or Other Pacific Islander, multiracial, and other race or ethnicity.

## Discussion

Using electronic health record data from more than 4.2 million ED encounters, this cross-sectional study was the first, to our knowledge, to explore the degree to which racial and ethnic inequity in physical restraint use may be mediated by police transport. Our analysis was consistent with previous findings that Black patients, compared with White patients, are more likely to be restrained in the ED in general,^6,12^ which may be related to a combination of inappropriate use of restraints in Black populations alongside inappropriate nonuse of restraints in other groups. In addition, our data suggest that Black patients were more likely to have been brought to the hospital via police transport, and that approximately 10.70% of the excess risk of restraint may have been mediated by police transport, even when adjusting for demographic and diagnostic factors.

There are a few possible explanations for the potentially mediating association between police transport of Black individuals and restraint use. First, Black individuals face higher rates of morbidity and mortality during interactions with police and may therefore experience police transport to be particularly criminalizing.^8,18^ Given that racial and ethnic minority communities have experienced a long history of institutional racism that warrants distrust of law enforcement,^19,20^ a criminalizing and inherently coercive experience such as being transported by police could lead to escalation of distress, psychomotor agitation, and behavioral decompensation, ultimately necessitating restraint. Second, the ED itself may be intrinsically carceral for those who are brought in by law enforcement, especially for Black patients experiencing mental health crises.^21^ Patients who are brought by police are often prohibited from leaving the ED voluntarily. In both Connecticut and North Carolina, police officers may, under certain circumstances, escort patients against their will to the ED for clinical evaluation.^22,23^^,^ In both states, and in most of the US, patients brought to the ED by law enforcement are often restricted to a locked unit, confined to their rooms, surveilled, and denied many belongings, such as their clothes, cell phones, and shoes. It is essential to recognize and address these carceral aspects of emergency care so that we can be thoughtful in understanding how patients of racial and ethnic minority and criminalized groups may be more acutely affected by them. Third, it is also possible that the disproportionate involvement of police in the transport of Black patients creates a perception of threat among the treatment team. Implicit or explicit biases among health care professionals and law enforcement could influence the risk-benefit decisions made by clinicians regarding the use of physical restraint.^24^ Medical resources might even be delayed based on a patient’s actual or perceived criminality.^25^ Finally, reduced access to outpatient medical treatment for racial and ethnic minority groups may lead to increased exposure to police during behavioral crises, warranting emergency evaluation and possible restraint use. Structural disadvantage has been associated with other chronic health conditions.^26^

Emerging data indicate that police are increasingly involved in the transport of patients to emergency medical services, and some municipalities have codified the practice of police transport for serious penetrating wounds.^7,27^ Although there could be legitimate reasons for police involvement in emergency medical transport, such as decreasing transport time for penetrating traumas, studies assessing outcomes of police-involved transport are limited, and results of these analyses have been mixed and largely restricted to populations with penetrating trauma.^28^ However, nearly half of the visits with police transport to emergency medical care may involve patients with mental illnesses, a group at particular risk for restraint, even without police involvement.^7^

Taken into context, our finding that police transport may mediate racially disparate restraint use in EDs highlights the need to further evaluate police involvement in emergency medical transport, especially in racial and ethnic minority communities and neighborhoods where there is continued reliance on law enforcement as first responders for mental health crises.^19^ Some have called for a reimagining or decoupling of policing from delivery of emergency medical services, particularly for racial and ethnic minority populations experiencing mental health crises.^29,30^ Advocates contend that unarmed clinicians should lead response teams for those experiencing mental health emergencies, which is consistent with the national guidelines for behavioral health crisis care, though it is unclear if such teams would mitigate disparities in restraint use.^29,31^ Additionally, others have advocated for separate units for those undergoing mental health emergencies; crisis stabilization units are built within or near EDs and could theoretically offer a calmer, less carceral environment for patients who may not require a hospital level of care for stabilization.^32,33,34^ However, there has been little research to compare efficacy of these units with that of classic EDs in addressing mental health emergencies.

To properly address disparities and inform policy around both patient transport and restraint use in EDs, future studies may assess recovery-oriented outcomes such as engagement with treatment, personal recovery, sense of empowerment, and quality of life following police transport to the hospital. Additionally, more work should be done to examine the interactions and intersections of race, sex, socioeconomic status, and diagnosis in police transportation and restraint use.

### Limitations

Our study has several limitations. First, its cross-sectional nature precluded assessment of causality. Due to the retrospective nature of the study, diagnosis codes assigned in the ED for psychiatric, medical, and substance use disorders may have been incomplete or inaccurate. Furthermore, we did not capture restraint data for patients with police escorts who arrived at the hospital in handcuffs or shackles and remained restrained during their visits. Our outcome was restraint use as a clinical intervention ordered under Centers for Medicare & Medicaid Services regulations. We also could not know the intent or appropriateness of restraint use. Given the nature of the dataset, we could not measure all possible confounders and mediators or assess data at the level of hospital site. As in all regional studies, the populations sampled for our analysis may not be generalizable to the whole population of the US. Finally, we acknowledge that evidence of mediation is hypothesis generating, not proof of causation. Nonetheless, mediation via police transport remained significant even with attempted adjustment for all potential confounding variables available in this dataset (eTable 2 in Supplement 1). Despite these limitations, this analysis was based on a large dataset across 2 regional health systems and adds important context to the growing body of literature on racially inequitable restraint use in emergency settings.

## Conclusions

In this cross-sectional study of over 4.2 million patient visits to 13 hospital EDs in the southeastern and northeastern US, we found that Black patients experienced a higher rate of police transport to the ED than White patients, potentially mediating a disproportionately higher rate of physical restraint during their ED visits. These results highlight possible intersectional disparities in behavioral emergency care. This analysis, as well as other emerging literature, may prompt clinicians to consider the ways in which restrictive safety interventions may influence disparate clinical outcomes for patients. Future studies promoting health justice in emergency and mental health care will require careful consideration of the pressures, particularly those related to systemic and structural racism, that shape the system of health care delivery.

## Notes

Supplement 1. **eTable 1.** Multivariable Logistic Regression Analyses for Associations Between Demographic and Visit Characteristics and Violent Restraint Use Including Police Transport as a Variable **eTable 2.** Post Hoc Interaction Analysis of Race and Ethnicity, Sex, and Age in Restraint Rates **eFigure.** Visualizing Interactions in Predicted Values of Violent Restraint by Age, Sex, and Race and Ethnicity

Supplement 2.
Data Sharing Statement
