# Supplementary material for: Racial and Ethnic Disparities in Patient Restraint in Emergency Departments by Police Transport Status
Source: JAMA Netw Open. 2024 Feb 21;7(2):e240098. doi: 10.1001/jamanetworkopen.2024.0098 (PMC10882414; doi:10.1001/jamanetworkopen.2024.0098)
Supplement: Supplement 1. — eTable 1. Multivariable Logistic Regression Analyses for Associations Between Demographic and Visit Characteristics and Violent Restraint Use Including Police Transport as a Variable eTable 2. Post Hoc Interaction Analysis of Race and Ethnicity, Sex, and Age in Restraint Rates eFigure. Visualizing Interactions in Predicted Values of Violent Restraint by Age, Sex, and Race and Ethnicity [file jamanetwopen-e240098-s001.pdf]

Supplementary Online Content

Chang-Sing E, Smith CM, Gagliardi JP, et al. Racial and ethnic disparities in patient restraint in emergency departments by police transport status. *JAMA Netw Open*. 2024;7(2):e240098. doi:10.1001/jamanetworkopen.2024.0098

eTable 1. Multivariable Logistic Regression Analyses for Associations Between Demographic and Visit Characteristics and Violent Restraint Use Including Police Transport as a Variable
eTable 2. Post Hoc Interaction Analysis of Race and Ethnicity, Sex, and Age in Restraint Rates
eFigure. Visualizing Interactions in Predicted Values of Violent Restraint by Age, Sex, and Race and Ethnicity

This supplementary material has been provided by the authors to give readers additional information about their work.

© 2024 Chang-Sing E et al. *JAMA Network Open*.

eTable 1. Multivariable Logistic Regression Analyses for Associations Between Demographic and Visit Characteristics and Violent Restraint Use Including Police Transport as a Variable

Demographic and Visit Characteristics	Nested unadjusted Odds Ratios		Nested adjusted Odds Ratios[a]	
	OR (95% CI)	p-value	OR (95% CI)	p-value
Sex				
Female	Ref.	Ref.	Ref.	Ref.
Male	2.37 (2.30 - 2.44)	< 0.001	1.49 (1.44-1.54)	< 0.001
Race/ethnicity				
Black Non-Hispanic	1.29 (1.25 - 1.33)	< 0.001	1.31 (1.27-1.35)	< 0.001
Hispanic/Latino	0.99 (0.95 - 1.03)	0.49	0.92 (0.88-0.96)	< 0.001
Other	0.85 (0.78 - 0.93)	< 0.001	1.15 (1.05-1.25)	< 0.001
Unknown	1.12 (0.99 - 1.28)	0.07	1.68 (1.47-1.92)	< 0.001
White Non-Hispanic	Ref.	Ref.	Ref.	Ref.
Age				
18-25	0.88 (0.84 - 0.92)	< 0.001	1.23 (1.17-1.29)	< 0.001
26-35	1.13 (1.09 - 1.18)	< 0.001	1.24 (1.18-1.29)	< 0.001
36-45	Ref.	Ref.	Ref.	Ref.
46-55	0.78 (0.75 - 0.82)	< 0.001	0.73 (0.70-0.77)	< 0.001
56-64	0.54 (0.51 - 0.57)	< 0.001	0.60 (0.57-0.64)	< 0.001
65-114	0.31 (0.29 - 0.32)	< 0.001	0.65 (0.61-0.68)	< 0.001
Previous Psychiatric History				
No	Ref.	Ref.	Ref.	Ref.
Yes	1.85 (1.92 - 6.58)	< 0.001	2.41 (2.31-2.52)	< 0.001
Medical Visit Diagnoses	0.21 (0.20 - 0.21)	< 0.001	0.47 (0.45-0.48)	< 0.001
Psychiatric Visit Diagnoses	12.6 (12.3 - 13.0)	< 0.001	3.37 (3.26-3.48)	< 0.001
Substance Related Visit Diagnoses	24.6 (23.9 - 25.3)	< 0.001	8.96 (8.62-9.30)	< 0.001
Trauma Visit Diagnoses	0.64 (0.61 - 0.67)	< 0.001	1.04 (0.99-1.09)	0.17
Cognitive/Neurologic Visit Diagnoses	2.06 (1.99 - 2.13)	< 0.001	2.19 (2.11-2.26)	< 0.001
Police Transport	23.23 (22.22 - 24.29)	< 0.001	5.51 (5.21-5.82)	< 0.001

[a] Adjusted for sex, race/ethnicity, age, site, previous psychiatric history, visit diagnoses, and police transport.

© 2024 Chang-Sing E et al. *JAMA Network Open*.

eTable 2. Post Hoc Interaction Analysis of Race and Ethnicity, Sex, and Age in Restraint Rates

Demographic and Visit Characteristics	Interaction Analysis	
	Estimate (Standard error)	p-value
Race/Ethnicity-Sex Interaction		
Black – Male	0.275 (0.033)	<0.001***
Hispanic/Latino – Male	0.467 (0.043)	<0.001***
Other – Male	0.145 (0.089)	0.104
Unknown – Male	0.343 (0.141)	0.014*
Race/Ethnicity-Age Interaction		
Black – 18-25 years	-0.054(0.054)	0.315
Hispanic/Latino – 18-25 years	0.154 (0.064)	0.016*
Other – 18-25 years	0.183 (0.126)	0.146
Unknown – 18-25 years	-0.209 (0.190)	0.270
Black – 26-35 years	-0.047 (0.047)	0.317
Hispanic/Latino – 26-35 years	-0.023 (0.058)	0.693
Other – 26-35 years	-0.104 (0.124)	0.399
Unknown – 26-35 years	-0.225 (0.181)	0.215
Black – 46-55 years	-0.271 (0.052)	<0.001***
Hispanic/Latino – 46-55 years	-0.036 (0.067)	0.586
Other – 46-55 years	-0.391 (0.158)	0.014
Unknown – 46-55 years	-0.445 (0.228)	0.051
Black – 56-64 years	-0.373 (0.063)	<0.001***
Hispanic/Latino – 56-64 years	0.019 (0.085)	0.820
Other – 56-64 years	-0.291 (0.199)	0.144
Unknown – 56-64 years	-0.649 (0.301)	0.031*
Black – 65+	0.030 (0.064)	0.644
Hispanic/Latino – 65+	0.422 (0.091)	<0.001***
Other – 65+	0.158 (0.182)	0.384
Unknown 65+	-0.091 (0.262)	0.730

© 2024 Chang-Sing E et al. *JAMA Network Open*.

eFigure. Visualizing Interactions in Predicted Values of Violent Restraint by Age, Sex, and Race and Ethnicity

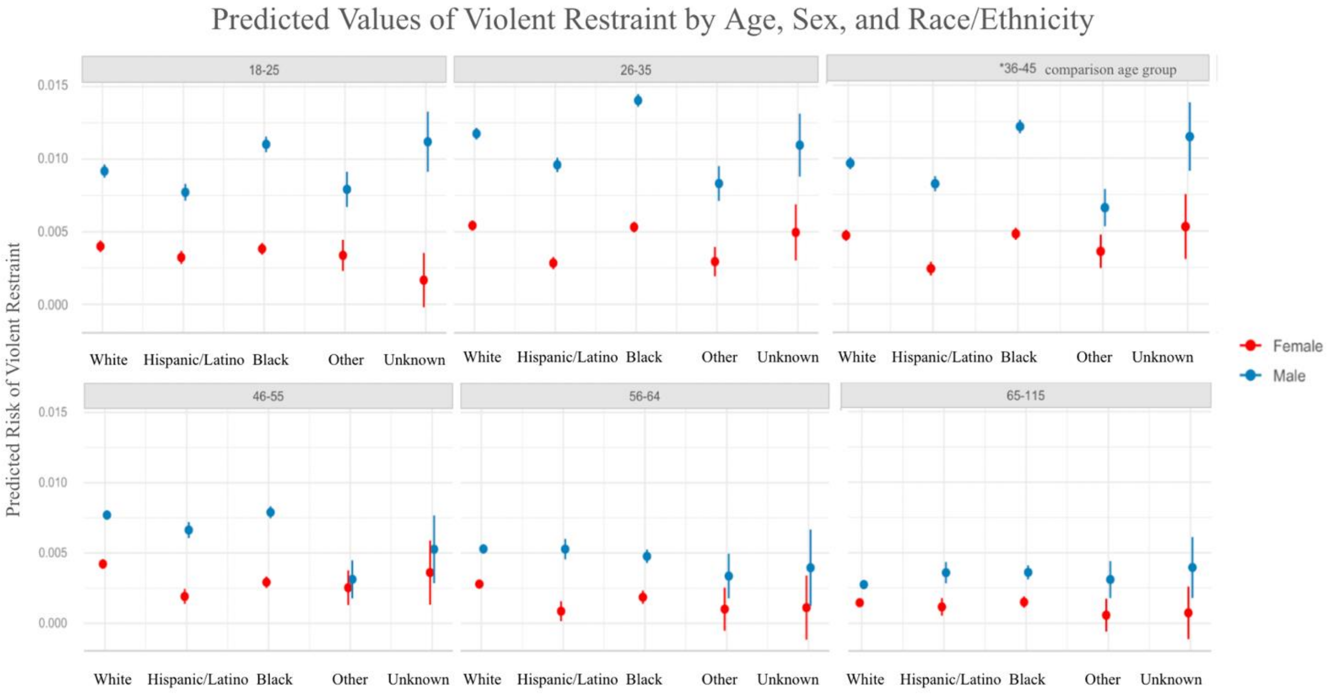

© 2024 Chang-Sing E et al. *JAMA Network Open*.
