# Supplementary material for: Racial and Ethnic Disparities in Patient Restraint in Emergency Departments by Police Transport Status
Source: JAMA Netw Open. 2024 Feb 21;7(2):e240098. doi: 10.1001/jamanetworkopen.2024.0098 (PMC10882414; doi:10.1001/jamanetworkopen.2024.0098)
Supplement: Supplement 2. — Data Sharing Statement [file jamanetwopen-e240098-s002.pdf]

Data Sharing Statement

Chang-Sing. Racial and Ethnic Disparities in Patient Restraint in Emergency Departments by Police Transport Status. *JAMA Netw Open*. Published February 21, 2024.
doi:10.1001/jamanetworkopen.2024.0098

Data
Data available: No
